# 4-(3,7-Dimethyl-4-oxo-4,5-dihydro­isoxazolo[4,5-*d*]pyridazin-5-yl)benzene­sulfonamide

**DOI:** 10.1107/S1600536811034325

**Published:** 2011-08-27

**Authors:** Abdullah M. Asiri, Hassan M. Faidallah, Abdulrahman O. Al-Youbi, Abdullah.Y. Obaid, Seik Weng Ng

**Affiliations:** a Chemistry Department, Faculty of Science, King Abdulaziz University, PO Box 80203 Jeddah, Saudi Arabia; b Department of Chemistry, University of Malaya, 50603 Kuala Lumpur, Malaysia

## Abstract

The nine-membered fused-ring system of the title pyridazine derivative, C_13_H_12_N_4_O_4_S, is approximately planar (r.m.s. deviation 0.027 Å), and the benzene ring of the phenyl­sulfamide substituent is aligned at 43.5 (1)° to the fused-ring system. The amine group of the sulfonamide substituent forms an N—H⋯O hydrogen bond to the ketonic O atom of two neigboring mol­ecules to generate a chain running along the *c* axis.

## Related literature

For a related structure, see: Abdel-Aziz *et al.* (2010 ▶). For the biological activity of the class of pyridazines, see: Faid-Allah *et al.* (2011 ▶); Makki & Faid-Allah (1996 ▶).
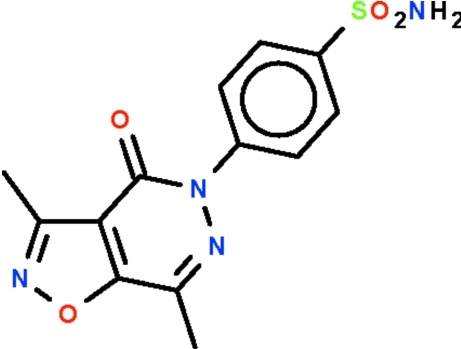


## Experimental

### 

#### Crystal data

C_13_H_12_N_4_O_4_S
*M* *_r_* = 320.33
Orthorhombic, 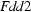

*a* = 18.0113 (4) Å
*b* = 35.5302 (11) Å
*c* = 8.2900 (2) Å
*V* = 5305.1 (2) Å^3^
*Z* = 16
Cu *K*α radiation
μ = 2.43 mm^−1^
*T* = 100 K
0.30 × 0.20 × 0.05 mm

#### Data collection

Agilent Technologies SuperNova Dual diffractometer with Atlas detector
Absorption correction: multi-scan (*CrysAlis PRO*; Agilent, 2010 ▶) *T*_min_ = 0.529, *T*_max_ = 0.888
7699 measured reflections
1886 independent reflections
1870 reflections with *I* > 2σ(*I*)
*R*_int_ = 0.032

#### Refinement

*R*[*F* ^2^ > 2σ(*F* ^2^)] = 0.033
*wR*(*F* ^2^) = 0.096
*S* = 1.08
1886 reflections
207 parameters
1 restraint
H atoms treated by a mixture of independent and constrained refinement
Δρ_max_ = 0.41 e Å^−3^
Δρ_min_ = −0.35 e Å^−3^
Absolute structure: Flack (1983 ▶), 441 Friedel pairs
Flack parameter: 0.026 (18)

### 

Data collection: *CrysAlis PRO* (Agilent, 2010 ▶); cell refinement: *CrysAlis PRO*; data reduction: *CrysAlis PRO*; program(s) used to solve structure: *SHELXS97* (Sheldrick, 2008 ▶); program(s) used to refine structure: *SHELXL97* (Sheldrick, 2008 ▶); molecular graphics: *X-SEED* (Barbour, 2001 ▶); software used to prepare material for publication: *publCIF* (Westrip, 2010 ▶).

## Supplementary Material

Crystal structure: contains datablock(s) global, I. DOI: 10.1107/S1600536811034325/xu5287sup1.cif

Structure factors: contains datablock(s) I. DOI: 10.1107/S1600536811034325/xu5287Isup2.hkl

Supplementary material file. DOI: 10.1107/S1600536811034325/xu5287Isup3.cml

Additional supplementary materials:  crystallographic information; 3D view; checkCIF report

## Figures and Tables

**Table 1:** Hydrogen-bond geometry (Å, °)

*D*—H⋯*A*	*D*—H	H⋯*A*	*D*⋯*A*	*D*—H⋯*A*
N4—H1⋯O2^i^	0.95 (3)	2.09 (4)	3.012 (3)	163 (3)
N4—H2⋯O2^ii^	0.85 (5)	2.11 (5)	2.933 (3)	162 (4)

Symmetry codes: (i) 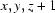; (ii) 
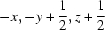
.

**Fig. 1.**
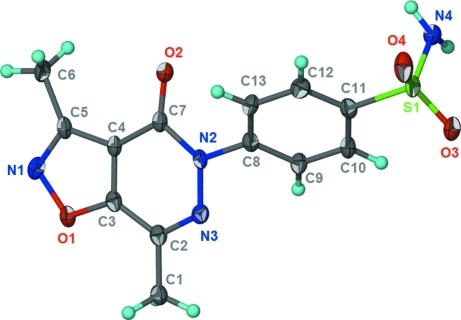
Thermal ellipsoid plot (Barbour, 2001) of C13H12N4O4S at the 70% probability level; hydrogen atoms are drawn as spheres of arbitrary radius.

## supplementary crystallographic information

### Comment

We have reported the synthesis of some pyridazines, which exhibit biological activity (Faid-Allah *et al.*, 2011; Makki & Faid-Allah, 1996). There are few crystal structure reports of such systems; recently, we reported the crystal structure of 3-methyl-2-(4-methyl)-2*H*-pyrazolo[3,4-*d*]pyridazin-5-ium thiocyanate, a salt (Abdel-Aziz *et al.*, 2010).

The nine-membered fused-ring system of C_13_H_12_N_4_O_4_S (Scheme I), is planar and the benzene ring of the phenylsufamido substitutent is aligned at 43.5 (1) ° (Fig. 1). The amino group the substitutent forms a hydrogen bond to the ketonic O atom of two neigboring molecules to generate a chain running along the *c*-axis of the orthorhombic unit cell (Table 1).

### Experimental

A solution of ethyl 5-acetyl-3-methylisoxazole-4-carboxylate (0.39 g, 0.002 mol) in ethanol (25 ml) was refluxed with *p*-sulfonamidophenyl hydrazine hydrochloride (0.49 g, 0.002 mol) for 2 h. The pyridazine which separated after concentration of the reaction mixture was filtered off, washed with ethanol and recrystallized from the same solvent to give long thin prisms in 90% yield, m.p. 488 K.

### Refinement

Carbon-bound H-atoms were placed in calculated positions [C—H 0.95 to 0.98 Å, *U*_iso_(H) 1.2 to 1.5*U*_eq_(C)] and were included in the refinement in the riding model approximation.

The amino H-atoms were located in a difference Fourier map, and were refined freely.

The Flack (Flack, 1983) parameter was refined from 441 Friedel pairs.

### Crystal data

**Table:**

C_13_H_12_N_4_O_4_S	*F*(000) = 2656
*M**_r_* = 320.33	*D*_x_ = 1.604 Mg m^−^^3^
Orthorhombic, *F**d**d*2	Cu *K*α radiation, λ = 1.54184 Å
Hall symbol: F 2 -2d	Cell parameters from 5872 reflections
*a* = 18.0113 (4) Å	θ = 4.9–74.4°
*b* = 35.5302 (11) Å	µ = 2.43 mm^−^^1^
*c* = 8.2900 (2) Å	*T* = 100 K
*V* = 5305.1 (2) Å^3^	Plate, colorless
*Z* = 16	0.30 × 0.20 × 0.05 mm

### Data collection

**Table:**

Agilent Technologies SuperNova Dual diffractometer with Atlas detector	1886 independent reflections
Radiation source: SuperNova (Cu) X-ray Source	1870 reflections with *I* > 2σ(*I*)
Mirror	*R*_int_ = 0.032
Detector resolution: 10.4041 pixels mm^-1^	θ_max_ = 74.5°, θ_min_ = 5.0°
ω scan	*h* = −42→44
Absorption correction: multi-scan (*CrysAlis PRO*; Agilent, 2010)	*k* = −22→22
*T*_min_ = 0.529, *T*_max_ = 0.888	*l* = −10→7
7699 measured reflections	

### Refinement

**Table:**

Refinement on *F*^2^	Secondary atom site location: difference Fourier map
Least-squares matrix: full	Hydrogen site location: inferred from neighbouring sites
*R*[*F*^2^ > 2σ(*F*^2^)] = 0.033	H atoms treated by a mixture of independent and constrained refinement
*wR*(*F*^2^) = 0.096	*w* = 1/[σ^2^(*F*_o_^2^) + (0.0772*P*)^2^ + 4.3892*P*] where *P* = (*F*_o_^2^ + 2*F*_c_^2^)/3
*S* = 1.08	(Δ/σ)_max_ < 0.001
1886 reflections	Δρ_max_ = 0.41 e Å^−^^3^
207 parameters	Δρ_min_ = −0.35 e Å^−^^3^
1 restraint	Absolute structure: Flack (1983), 441 Friedel pairs
Primary atom site location: structure-invariant direct methods	Flack parameter: 0.026 (18)

### Special details

**Table:**

Geometry. All esds (except the esd in the dihedral angle between two l.s. planes) are estimated using the full covariance matrix. The cell esds are taken into account individually in the estimation of esds in distances, angles and torsion angles; correlations between esds in cell parameters are only used when they are defined by crystal symmetry. An approximate (isotropic) treatment of cell esds is used for estimating esds involving l.s. planes.

### Fractional atomic coordinates and isotropic or equivalent isotropic displacement parameters (Å^2^)

**Table:**

	*x*	*y*	*z*	*U*_iso_*/*U*_eq_	
S1	0.12413 (3)	0.212823 (15)	0.43880 (7)	0.01769 (17)	
O1	0.21513 (9)	0.37146 (4)	−0.5296 (2)	0.0193 (4)	
O2	0.05983 (9)	0.28881 (4)	−0.2671 (2)	0.0188 (4)	
O3	0.17529 (10)	0.22599 (5)	0.5595 (2)	0.0235 (4)	
N4	0.04217 (11)	0.22369 (5)	0.5034 (3)	0.0213 (4)	
N1	0.14841 (11)	0.36838 (5)	−0.6234 (3)	0.0200 (4)	
N2	0.17467 (10)	0.30278 (5)	−0.1592 (3)	0.0154 (4)	
N3	0.24011 (10)	0.32316 (5)	−0.1526 (3)	0.0163 (4)	
O4	0.12339 (11)	0.17367 (5)	0.3966 (3)	0.0277 (5)	
C1	0.32798 (13)	0.36781 (6)	−0.2608 (3)	0.0204 (5)	
H1A	0.3547	0.3612	−0.1618	0.031*	
H1B	0.3168	0.3948	−0.2603	0.031*	
H1C	0.3589	0.3618	−0.3546	0.031*	
C2	0.25679 (12)	0.34585 (6)	−0.2694 (3)	0.0170 (5)	
C3	0.20621 (12)	0.34980 (6)	−0.3997 (3)	0.0169 (5)	
C4	0.13880 (13)	0.33186 (6)	−0.4030 (3)	0.0156 (5)	
C5	0.10469 (13)	0.34528 (6)	−0.5465 (3)	0.0177 (5)	
C6	0.02971 (13)	0.33542 (7)	−0.6145 (3)	0.0213 (5)	
H6A	0.0218	0.3492	−0.7153	0.032*	
H6B	−0.0090	0.3423	−0.5367	0.032*	
H6C	0.0275	0.3083	−0.6357	0.032*	
C7	0.11926 (12)	0.30620 (6)	−0.2766 (3)	0.0155 (5)	
C8	0.16360 (12)	0.28011 (6)	−0.0177 (3)	0.0159 (5)	
C9	0.18337 (13)	0.29491 (6)	0.1314 (3)	0.0175 (5)	
H9	0.2040	0.3195	0.1383	0.021*	
C10	0.17290 (12)	0.27382 (6)	0.2696 (3)	0.0175 (5)	
H10	0.1876	0.2835	0.3715	0.021*	
C11	0.14063 (12)	0.23826 (6)	0.2583 (3)	0.0171 (5)	
C12	0.12297 (12)	0.22293 (7)	0.1099 (4)	0.0189 (5)	
H12	0.1026	0.1983	0.1037	0.023*	
C13	0.13496 (12)	0.24347 (6)	−0.0300 (3)	0.0174 (5)	
H13	0.1240	0.2329	−0.1325	0.021*	
H1	0.0433 (17)	0.2472 (9)	0.559 (4)	0.025 (8)*	
H2	0.008 (2)	0.2165 (10)	0.439 (6)	0.043 (10)*	

### Atomic displacement parameters (Å^2^)

**Table:**

	*U*^11^	*U*^22^	*U*^33^	*U*^12^	*U*^13^	*U*^23^
S1	0.0235 (3)	0.0179 (3)	0.0117 (3)	−0.00015 (18)	−0.0010 (2)	0.0027 (2)
O1	0.0226 (8)	0.0227 (7)	0.0124 (9)	−0.0015 (6)	0.0005 (7)	0.0035 (7)
O2	0.0198 (8)	0.0231 (7)	0.0135 (9)	−0.0028 (5)	−0.0009 (7)	0.0002 (7)
O3	0.0273 (9)	0.0289 (8)	0.0143 (10)	−0.0008 (7)	−0.0034 (8)	0.0049 (7)
N4	0.0219 (10)	0.0270 (10)	0.0150 (11)	−0.0041 (8)	−0.0011 (9)	0.0002 (9)
N1	0.0230 (9)	0.0225 (9)	0.0145 (11)	0.0025 (7)	−0.0033 (9)	0.0000 (8)
N2	0.0190 (8)	0.0174 (8)	0.0098 (10)	−0.0005 (7)	0.0018 (8)	0.0000 (8)
N3	0.0183 (9)	0.0169 (7)	0.0136 (10)	0.0005 (7)	0.0002 (8)	−0.0033 (8)
O4	0.0464 (11)	0.0194 (8)	0.0172 (11)	0.0006 (7)	−0.0003 (8)	0.0042 (8)
C1	0.0236 (10)	0.0242 (10)	0.0134 (13)	−0.0058 (8)	0.0020 (11)	−0.0016 (9)
C2	0.0208 (10)	0.0163 (8)	0.0139 (12)	0.0009 (8)	0.0016 (10)	−0.0015 (8)
C3	0.0219 (10)	0.0166 (9)	0.0123 (12)	0.0003 (8)	0.0025 (10)	−0.0015 (9)
C4	0.0201 (10)	0.0171 (9)	0.0096 (12)	0.0017 (8)	0.0007 (9)	−0.0015 (9)
C5	0.0220 (10)	0.0202 (9)	0.0107 (12)	0.0031 (8)	0.0020 (10)	0.0003 (9)
C6	0.0220 (11)	0.0291 (11)	0.0128 (12)	0.0017 (8)	−0.0028 (10)	0.0024 (10)
C7	0.0195 (10)	0.0164 (9)	0.0107 (13)	0.0032 (7)	0.0005 (9)	−0.0007 (9)
C8	0.0170 (9)	0.0176 (9)	0.0130 (13)	0.0022 (7)	0.0013 (10)	0.0015 (9)
C9	0.0220 (10)	0.0176 (10)	0.0127 (12)	−0.0001 (8)	0.0001 (10)	−0.0016 (9)
C10	0.0223 (10)	0.0195 (10)	0.0107 (11)	0.0017 (8)	0.0003 (10)	−0.0007 (9)
C11	0.0179 (9)	0.0186 (10)	0.0147 (13)	0.0027 (8)	0.0016 (10)	0.0030 (10)
C12	0.0210 (11)	0.0171 (9)	0.0186 (14)	0.0000 (8)	−0.0009 (10)	−0.0022 (10)
C13	0.0212 (10)	0.0189 (10)	0.0122 (13)	0.0004 (8)	−0.0022 (9)	−0.0023 (9)

### Geometric parameters (Å, °)

**Table:**

S1—O4	1.4345 (19)	C2—C3	1.420 (3)
S1—O3	1.4385 (19)	C3—C4	1.372 (3)
S1—N4	1.617 (2)	C4—C5	1.422 (4)
S1—C11	1.773 (3)	C4—C7	1.433 (3)
O1—C3	1.333 (3)	C5—C6	1.505 (3)
O1—N1	1.435 (3)	C6—H6A	0.9800
O2—C7	1.238 (3)	C6—H6B	0.9800
N4—H1	0.95 (3)	C6—H6C	0.9800
N4—H2	0.85 (5)	C8—C9	1.389 (4)
N1—C5	1.304 (3)	C8—C13	1.404 (3)
N2—N3	1.384 (3)	C9—C10	1.382 (3)
N2—C7	1.399 (3)	C9—H9	0.9500
N2—C8	1.437 (3)	C10—C11	1.394 (3)
N3—C2	1.296 (3)	C10—H10	0.9500
C1—C2	1.502 (3)	C11—C12	1.383 (4)
C1—H1A	0.9800	C12—C13	1.387 (4)
C1—H1B	0.9800	C12—H12	0.9500
C1—H1C	0.9800	C13—H13	0.9500
			
O4—S1—O3	119.41 (11)	N1—C5—C4	111.0 (2)
O4—S1—N4	107.69 (11)	N1—C5—C6	120.4 (2)
O3—S1—N4	106.07 (12)	C4—C5—C6	128.5 (2)
O4—S1—C11	106.85 (12)	C5—C6—H6A	109.5
O3—S1—C11	108.30 (11)	C5—C6—H6B	109.5
N4—S1—C11	108.10 (11)	H6A—C6—H6B	109.5
C3—O1—N1	107.01 (16)	C5—C6—H6C	109.5
S1—N4—H1	110.4 (18)	H6A—C6—H6C	109.5
S1—N4—H2	112 (3)	H6B—C6—H6C	109.5
H1—N4—H2	125 (3)	O2—C7—N2	121.9 (2)
C5—N1—O1	106.8 (2)	O2—C7—C4	125.2 (2)
N3—N2—C7	126.1 (2)	N2—C7—C4	112.9 (2)
N3—N2—C8	112.27 (18)	C9—C8—C13	120.6 (2)
C7—N2—C8	121.16 (18)	C9—C8—N2	118.59 (18)
C2—N3—N2	119.6 (2)	C13—C8—N2	120.8 (2)
C2—C1—H1A	109.5	C10—C9—C8	119.8 (2)
C2—C1—H1B	109.5	C10—C9—H9	120.1
H1A—C1—H1B	109.5	C8—C9—H9	120.1
C2—C1—H1C	109.5	C9—C10—C11	119.5 (2)
H1A—C1—H1C	109.5	C9—C10—H10	120.2
H1B—C1—H1C	109.5	C11—C10—H10	120.2
N3—C2—C3	118.8 (2)	C12—C11—C10	120.8 (2)
N3—C2—C1	119.0 (2)	C12—C11—S1	120.77 (17)
C3—C2—C1	122.2 (2)	C10—C11—S1	118.4 (2)
O1—C3—C4	111.0 (2)	C11—C12—C13	120.0 (2)
O1—C3—C2	126.5 (2)	C11—C12—H12	120.0
C4—C3—C2	122.5 (2)	C13—C12—H12	120.0
C3—C4—C5	104.1 (2)	C12—C13—C8	119.0 (2)
C3—C4—C7	119.9 (2)	C12—C13—H13	120.5
C5—C4—C7	136.0 (2)	C8—C13—H13	120.5
			
C3—O1—N1—C5	−0.6 (2)	C3—C4—C7—O2	179.4 (2)
C7—N2—N3—C2	−5.6 (3)	C5—C4—C7—O2	−1.1 (4)
C8—N2—N3—C2	−177.91 (19)	C3—C4—C7—N2	0.3 (3)
N2—N3—C2—C3	1.5 (3)	C5—C4—C7—N2	179.9 (2)
N2—N3—C2—C1	179.99 (19)	N3—N2—C8—C9	38.9 (3)
N1—O1—C3—C4	1.5 (2)	C7—N2—C8—C9	−133.9 (2)
N1—O1—C3—C2	−176.5 (2)	N3—N2—C8—C13	−139.7 (2)
N3—C2—C3—O1	−179.1 (2)	C7—N2—C8—C13	47.5 (3)
C1—C2—C3—O1	2.4 (4)	C13—C8—C9—C10	−1.8 (3)
N3—C2—C3—C4	3.2 (3)	N2—C8—C9—C10	179.60 (19)
C1—C2—C3—C4	−175.3 (2)	C8—C9—C10—C11	−1.8 (3)
O1—C3—C4—C5	−1.7 (2)	C9—C10—C11—C12	3.8 (3)
C2—C3—C4—C5	176.3 (2)	C9—C10—C11—S1	−177.18 (17)
O1—C3—C4—C7	177.95 (19)	O4—S1—C11—C12	24.3 (2)
C2—C3—C4—C7	−4.0 (3)	O3—S1—C11—C12	154.11 (18)
O1—N1—C5—C4	−0.5 (3)	N4—S1—C11—C12	−91.4 (2)
O1—N1—C5—C6	−179.15 (19)	O4—S1—C11—C10	−154.73 (18)
C3—C4—C5—N1	1.3 (3)	O3—S1—C11—C10	−24.9 (2)
C7—C4—C5—N1	−178.2 (2)	N4—S1—C11—C10	89.6 (2)
C3—C4—C5—C6	179.9 (2)	C10—C11—C12—C13	−2.2 (3)
C7—C4—C5—C6	0.3 (4)	S1—C11—C12—C13	178.81 (17)
N3—N2—C7—O2	−174.6 (2)	C11—C12—C13—C8	−1.4 (3)
C8—N2—C7—O2	−2.9 (3)	C9—C8—C13—C12	3.4 (3)
N3—N2—C7—C4	4.5 (3)	N2—C8—C13—C12	−178.04 (19)
C8—N2—C7—C4	176.19 (19)		

### Hydrogen-bond geometry (Å, °)

**Table:**

*D*—H···*A*	*D*—H	H···*A*	*D*···*A*	*D*—H···*A*
N4—H1···O2^i^	0.95 (3)	2.09 (4)	3.012 (3)	163 (3)
N4—H2···O2^ii^	0.85 (5)	2.11 (5)	2.933 (3)	162 (4)

Symmetry codes: (i) *x*, *y*, *z*+1; (ii) −*x*, −*y*+1/2, *z*+1/2.
